# The need for novel influenza vaccines in low- and middle-income countries: A narrative review

**DOI:** 10.1016/j.bjid.2024.104465

**Published:** 2024-12-05

**Authors:** Julia R. Spinardi, Karan B. Thakkar, Verna L. Welch, Oladayo Jagun, Moe H. Kyaw

**Affiliations:** a Vaccine Medical and Scientific Affairs, Emerging Markets, Pfizer Inc., São Paulo, SP, Brazil; b Vaccine Medical and Scientific Affairs, Emerging Markets, Pfizer Inc., Singapore, Singapore; c Vaccine Medical and Scientific Affairs, Pfizer Inc., Pennsylvania, USA; d Real World Strategy and Innovation, IQVIA Inc., New Jersey, USA; e Vaccine Medical and Scientific Affairs, Emerging Markets, Pfizer Inc., Virginia, USA

**Keywords:** Influenza, Immunization, Public health, Disease burden, Low- and middle-income countries

## Abstract

Influenza viruses cause 3–5 million severe cases and 300,000–600,000 deaths worldwide. Most of the disease burden is in Low- and Middle-Income Countries (LMICs) owing to factors such as high population density, infrastructure challenges, poor quality healthcare, lack of consistent recommendations, less prioritization of all high-risk groups, and prevalent use of trivalent influenza vaccines. Although influenza vaccines are effective in reducing the annual influenza disease burden, existing vaccines have several limitations. In this narrative review, we address the unmet needs of existing influenza vaccines in LMICs in Africa, Asia Pacific, Latin America and the Middle East and discuss the characteristics of novel vaccines in clinical development. We also describe features of a successful vaccination program that LMICs could emulate to improve their current vaccination coverage and reduce the public health burden of influenza.

## Introduction

Influenza is an important global public health threat, particularly in highly populated Low- and Middle-Income Countries (LMICs). Globally, seasonal influenza has been estimated to result in 290,000–650,000 deaths from respiratory causes^1^ and 99,000–200,000 deaths from Lower Respiratory Tract Infections (LRTIs) every year.^2^ In LMICs, seasonal influenza is estimated to account for a combined 83 % of the total annual influenza-related respiratory deaths worldwide.^1^ In terms of influenza-specific LRTIs, the wider Asia-Pacific region (excluding High Income Countried [HIC]) had the highest number of influenza-related LRTI deaths across all ages in 2017, representing 40 % of total LRTI deaths globally.^2^ Nonetheless, the actual influenza mortality burden is likely much higher when accounting for complications beyond respiratory illness, such as exacerbations of underlying chronic illness and increased susceptibility to Cardiovascular (CV) events and CV mortality.^3^

Vaccination remains the most effective strategy to reduce the disease burden of seasonal influenza and its complications. Currently approved seasonal influenza vaccines are effective against severe influenza and have demonstrated favorable safety profiles.^4^ Although influenza vaccines have been widely available for decades, a stark disparity remains in vaccine access between regions/countries. For instance, of the 531 million vaccine doses distributed worldwide in 2019, the World Health Organization (WHO) regions of the Americas and Europe accounted for 76 % of the distributed doses.^5^ Meanwhile, about three-quarters of the world's population residing in the remaining four WHO regions received a combined 24 % of the distributed doses in 2019.^5^ Inadequate influenza surveillance infrastructures, low vaccination rates, global inequity to vaccine access, inappropriate vaccination timings (particularly in tropical countries), high population densities and limited access to healthcare services can all exacerbate influenza burden in LMICs.

The LMICs of East Asia and the Pacific, Europe and Central Asia, Latin America and the Caribbean, North Africa and the Middle East, South Asia, and Sub-Saharan Africa were home to about 84 % of the world's population in 2021.^6^ Overall, these countries account for 38 % of the global gross domestic product.^6^ With increasing global economic importance, the overall burden of influenza in LMICs could present a huge social and economic impact on individuals, healthcare systems and society. This article describes the disease burden of influenza in LMICs, reviews the efficacy/effectiveness and safety of current influenza vaccines, and discusses the need for novel influenza vaccine platforms by outlining the unmet needs related to current vaccines and the characteristics of novel vaccines in clinical development.

### Overview of influenza virus biology

Influenza viruses belong to the *Orthomyxoviridae* family, including the clinically relevant influenza A and B viruses.^4^ Influenza A and B viruses are relatively simple, consisting of a viral envelope, matrix protein and the virion core.^7^

The genome of influenza A and B viruses comprises eight negative-sense, single-stranded viral RNA segments, coated with multiple copies of Nucleoproteins (NPs) and bound by an RNA-dependent RNA polymerase heterotrimeric complex, forming the Ribonucleoprotein (RNP) complex.^7^ In addition to the RNP complex, the virion core also includes nuclear export and nonstructural proteins.^7^

The core is enclosed within the Matrix protein (M1) and the glycoprotein-studded phospholipid bilayer envelope.^7^ The viral envelope anchors the Hemagglutinin (HA) and Neuraminidase (NA) glycoproteins – the predominant viral surface proteins that act as key regulators of viral entry and exit from host cells and are thus immunologically important antigens.^7^ There are 18 HA subtypes and 11 NA subtypes that have been identified in influenza to date.^8^ In addition, the viral envelope is also associated with the M2 protein that forms a tetrameric ion channel.^7^

### Antigenic evolution and implications for vaccination

Influenza viruses constantly evolve at their surface glycoproteins via antigenic drift or shift, allowing the virus to remain a threat to the human population despite repeated exposure and immunity to prior variants.^8^

Antigenic drift is the process by which point mutations in the viral genome introduce minor changes into viral epitopes.^8^ In the influenza virus genome, frequent mutations are introduced during the replication cycle owing to the lack of proofreading mechanisms associated with viral RNA-dependent RNA polymerases.^8^ Viruses with antigenic drift are reported to preferentially escape preexisting immunity.^8^ Antigenic drift is the main cause of seasonal epidemics and is responsible for the need to update vaccine composition every influenza season.^9^

Antigenic shift results in an exchange of HA genes, NA genes or both.^8^ The emergence of such a novel strain could result in a global pandemic.^8^ The capacity of the influenza A virus to cause a pandemic is related to its genetic diversity and ability to switch host species.^8^ There have been four pandemics in the last 100 years: the pandemics of 1918, 1957, 1968 and 2009.^8^^,^^9^

At present, the A(H3N2), 2009 A(H1N1) and influenza B viruses circulate seasonally among humans, causing substantial morbidity and mortality.^8^ Vaccinating against circulating viruses is the best approach to reducing the seasonal influenza disease burden.^4^

### Burden of seasonal influenza in LMICs

Seasonal influenza A and B cause significant disease and mortality, especially among unvaccinated older adults (aged ≥ 65 years) and young children (aged < 5 years).^4^ In general, influenza A poses a higher disease burden than influenza B; a recent study estimated that 23 % of all annual influenza cases are due to influenza B virus.^10^ Influenza B can be more common and severe than influenza A in children, causing up to 52 % of all influenza-related deaths.^11^

A study by Iuliano and colleagues revealed that older adults living in LMICs had the highest proportion of influenza-related respiratory deaths, accounting for 77 % of deaths within this age group.^1^ Among adults aged < 65 years, up to 93 % of influenza-related respiratory mortality occurred in LMICs.^1^ In WHO geographic regions (regardless of income level), older adults residing in the Western Pacific and South-East Asia regions had the highest proportion of influenza-related respiratory mortality, accounting for 57 % of deaths within this age group.^1^ Meanwhile, Sub-Saharan Africa and Eastern Mediterranean had the lowest proportion of influenza-related respiratory mortality within this age bracket.^1^

A 2018 study showed a substantial proportion of influenza-related burden in children aged 0–59 months. Of the estimated 786,000 LRTI total hospital admissions in young children (the burden estimates were calculated by summing up estimates in three non-overlapping age groups and three income levels according to the World Bank classification), LMICs contributed about 86 % of admissions.^12^ Of about 20,800 in-hospital deaths in young children, up to 98 % occurred among infants under 6 months living in LMICs.^12^ Unsurprisingly, the in-hospital case fatality ratio among young children was higher in LMICs at 4.1 % than in HICs at 0.5 %.^12^

Post-acute conditions have also been reported after influenza infection. Evidence shows that influenza infection could trigger acute Myocardial Infarction (MI) and stroke.^13^ Specifically, a higher incidence ratio for acute MI was observed after influenza infection in individuals aged > 65 years than in those aged ≤ 65 years (7.31 vs. 2.38).^14^ A study conducted in Beijing from 2011 to 2018 to assess the excess Cardiovascular Disease (CVD) mortality attributable to influenza in older adults estimated the annual excess mortality rate per 100,000 population at 27–49 for ischemic heart disease, 14–22 for ischemic stroke and 54–96 for overall CVD.^15^ The excess mortality was estimated to result in 916–1640 influenza-related CVD deaths among those aged ≥ 65 years,^15^ further reinforcing the high actual burden of influenza.

Seasonal influenza also exacts a heavy socioeconomic toll on healthcare systems and society. The proportion of costs attributable to influenza is particularly high among working adults, primarily as a result of workplace absence, reduced patient and caregiver productivity, and increased healthcare resource utilization. A study in South Africa from 2013 to 2015 estimated the mean annual total cost of influenza‐associated illness at USD 270.5 million, with indirect costs accounting for 44 % of the total cost.^16^ Similarly, a systematic review of 11 studies conducted in Thailand estimated the total cost of influenza at USD 31.1–83.6 million per year, with 50 %–53 % of the economic burden attributable to lost productivity.^17^

### Global influenza surveillance programs

An influenza surveillance system is indispensable in monitoring influenza epidemiology and disease burden. The WHO Global Influenza Surveillance and Response System (GISRS) – through a network of national influenza centers, collaborating centers and reference laboratories worldwide – is especially critical in the surveillance, detection, preparedness and response to pandemic influenza and informing forecasting for strain composition for seasonal influenza vaccines.^18^

The Global Initiative on Sharing All Influenza Data (GISAID) was later established to promote the rapid sharing of genetic and associated influenza virus data.^19^ The repository data is particularly valuable to help detect emerging strains and guide the selection of vaccine strains.^19^ More recently, the Global Influenza Hospital Surveillance Network (GIHSN) was developed to study the burden of severe influenza and estimate the effectiveness of seasonal influenza vaccines across seasons in different countries.^20^ The network uses a standard protocol and a common approach to case selection for testing and data selection, thereby avoiding potential biases.^20^

The WHO's Global Influenza Strategy 2019–2030 highlights the need to strengthen global influenza surveillance and monitoring. The document also called for a better understanding of the disease and its economic burden in LMICs, to guide the establishment or expansion of seasonal influenza prevention and control strategies.^21^ Despite influenza surveillance systems being a key component to successful influenza control and prevention, there is a clear disparity across WHO member states, particularly between HICs and LMICs.^22^ A 2021 study comparing national influenza surveillance systems revealed considerable variability between three Asia Pacific countries with different levels of development, with Australia demonstrating the broadest scope and most detailed influenza surveillance, followed by China and Malaysia.^22^

**Fig. 1:**
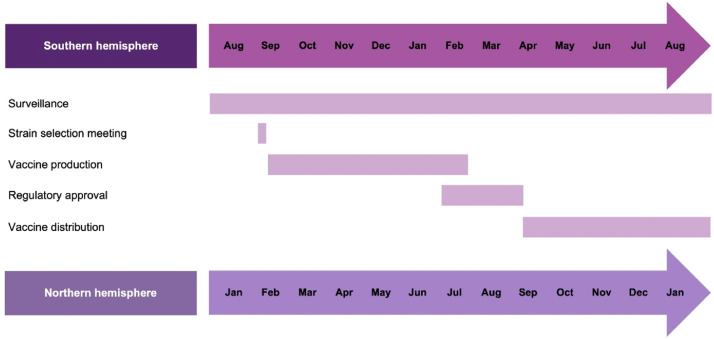
WHO timeline for vaccine strain selection meeting and manufacturing cycle.^24^ Analytical and Bioanalytical Chemistry. Antigenic characterization of influenza and SARS-CoV-2 viruses. Volume 414, 2021, 2841–2881, Wang Y, Tang CY, Wan XF. (Copyright© Springer-Verlag GmbH Germany, part of Springer Nature 2021) "With permission of Springer".

The WHO's Global Influenza Strategy 2019–2030 also encourages LMICs to use innovative modeling and data sources to improve the forecasting of influenza seasonality, inform influenza vaccine strain selection and recommend appropriate vaccination timing.^21^ Analysis of FluNet data from 2011 to 2016 confirmed that temperate regions of the northern and southern hemispheres showed a distinct seasonal peak during their respective winters, while influenza activity in tropical and sub-tropical countries could be seen throughout the year in various patterns.^23^ The dichotomy of influenza seasonality has been used to guide the vaccine strain selection meeting and manufacturing cycle (Fig. 1)^24^ and inform vaccination strategies.^23^ Currently, the GISRS scientific committee convenes a meeting twice a year (in February for the northern hemisphere and in September for the southern hemisphere) to provide recommendations on vaccine strains for the next influenza season.^24^

### Characteristics of currently licensed influenza vaccines

The three types of influenza vaccines currently approved for use include Inactivated (IIV), Live-Attenuated (LAIV) and Recombinant (RIV) influenza vaccines (Table 1).^25^ All three vaccine platforms are available as trivalent (containing two influenza A subtypes and one influenza B lineage) or quadrivalent (containing two influenza A subtypes and two influenza B lineages) formulations.

**Table 1:** Comparisons between influenza vaccine types currently approved for use.^25^

	Vaccine type	Manufacturing technology	Adjuvant	Administration route	Age indication^a^
**IIV4**	Subunit	Egg	None	IM/SC	≥ 6 months
		Egg	MF59	IM	≥ 65 years
		Cell culture	None	IM	≥ 4 years
	Split	Egg	None	IM/SC	≥ 6 months
	Split/high dose	Egg	None	IM	≥ 65 years
**IIV3**	Subunit	Egg	MF59	IM	≥ 65 years
**LAIV4**	Cold, adapted live virus	Egg	None	IN spray	2–49 years
**RIV4**	Recombinant HA	Cell culture	None	IM	≥ 18 years

a Please refer to product-specific, FDA-approved prescribing information for the most complete and updated information, as indications may vary by formulation. FDA, Food and Drug Administration; HA, Hemagglutinin; IIV3, Trivalent Inactivated Influenza Vaccine; IIV4, Quadrivalent Inactivated Influenza Vaccine; IM, Intramuscular; IN, Intranasal; LAIV4, Quadrivalent Live-Attenuated Influenza Vaccine; RIV4, Quadrivalent Recombinant Influenza Vaccine; SC, Subcutaneous.

Influenza vaccines are traditionally produced using Candidate Vaccine Viruses (CVVs) grown in embryonated chicken eggs.^26^ At present, almost all seasonal influenza vaccines are produced using egg-based technology due to the infrastructure availability and vast manufacturing requirement to meet the global annual demand for seasonal influenza vaccines.^26^ While egg-based vaccines are generally more cost-effective than their egg-free alternatives, the reliance on eggs makes them susceptible to egg supply shortage.^26^ Furthermore, the production of an egg-based vaccine can take about 6 months, giving time for antigenic drift away from CVVs to occur in circulating strains.^24^ Additionally, egg-adaptation changes in the virus could affect antigenicity and reduce Vaccine Effectiveness (VE) by an estimated 4 %–16 %.^27^ Moreover, egg-based vaccines are contraindicated in individuals with a history of severe allergic reactions to eggs.^26^

One way to improve the traditional egg-based influenza vaccines involves delivering a higher dose of viral antigens (i.e., four-fold higher than the standard vaccine) or incorporating adjuvants (such as emulsion-based MF59 adjuvant) into the vaccine formulation.^26^ Both strategies can enhance immune responses in older adults or individuals with weaker immune system, leading to improved VE.^26^ However, concerns related to egg-based technology remain.

Egg-free production methods, using cell-based or recombinant technologies, have also been developed and approved to overcome some drawbacks of egg-based vaccines. Cell-based technology involves growing CVVs in mammalian cells, while the more innovative RIV is produced using baculovirus vectors expressing recombinant HA protein subunit in insect cells.^26^ Neither approach uses eggs; this insulates them from egg supply shortages, egg-adaptive mutations and egg allergies.^26^

### Current influenza vaccines: efficacy and safety profiles

The overall efficacy and/or effectiveness of licensed influenza vaccines varies each year and depends on several key factors, with estimates ranging between 19 % and 52 % from 2011 through 2022 in the US.^28^ A meta-analysis conducted in China also revealed a moderate overall VE of 36 % from 2010 through 2018.^29^ Key factors affecting the efficacy (based on Randomized Controlled Trials [RCTs]) and/or effectiveness (based on real-world data) of influenza vaccines include recipient age and health status,^28^ vaccine manufacturing process^27^ and degree of strain match in an influenza season.^30^^,^^31^

#### Non-pregnant healthy adults

Evidence suggests that influenza vaccination is effective in reducing morbidity and mortality in non-pregnant healthy adults. A 2018 Cochrane review that included 52 RCTs of over 80,000 healthy adults showed that IIVs and LAIVs were similarly effective in reducing the incidence of Laboratory-Confirmed Influenza (LCI) and Influenza-Like Illness (ILI).^32^ Compared with the control group, receipt of IIVs led to 59 % and 16 % reductions in LCI and ILI, respectively.^32^ Likewise, LAIVs demonstrated a corresponding 53 % and 10 % effectiveness against LCI and ILI versus the control group.^32^ It is noteworthy that the effects of IIVs and LAIVs in preventing LCI or ILI were not significantly affected by vaccine match.^32^ Vaccine content also appeared not to affect the performance of LAIVs, unlike IIVs, in preventing ILI. However, IIVs only led to small reductions in influenza-related work absence.^32^

Studies evaluating the effect of vaccination in reducing influenza-related hospitalization revealed a greater impact on Intensive Care Unit (ICU) admission and death than on overall hospitalization.^33^ In the 2018 Cochrane review, IIVs resulted in small reductions in influenza-related hospitalization among healthy adults.^32^ A 2021 meta-analysis showed that vaccination did not significantly reduce the risk of hospitalization following outpatient influenza illness or clinical diagnosis of pneumonia among hospitalized adults.^33^ However, vaccination with current IIVs reduced the odds of ICU admission by 26 % among community-dwelling adults hospitalized for influenza.^33^ Crucially, vaccinated patients had 31 % less risk of influenza-related mortality than unvaccinated patients.^33^

#### Older adults

Real-world data have shown that influenza vaccines are generally less effective in older adults than in their younger counterparts.^28^ However, a 2018 Cochrane review of eight RCTs involving over 5000 older adults found that vaccination reduced LCI by 58 % and ILI by 41 %,^34^ higher than reported in healthy adults.^32^ Overall, the trials included in this Cochrane review did not show a significant vaccination effect against other measured outcomes, including all-cause mortality.^34^ In contrast, an earlier meta-analysis of 14 cohort studies found that vaccination effectively reduced all-cause mortality by 36 % (after adjusting for potential biases) among community-dwelling older adults.^35^ Similarly, a study in Brazil found that the introduction of an annual nationwide influenza vaccination program was associated with a 26 % reduction in overall mortality in older adults aged ≥ 65 years.^36^

Of note, VE estimates for preventing LCI and ILI during an outbreak season among community dwellers were lower than that observed with institutionalized older adults (59 % and 13 %–43 % vs. 65 % and 46 %–65 %, respectively).^34^ However, the findings of two RCTs revealed comparable VE in preventing ILI among community-dwelling adults aged ≥ 60 years in Thailand (VE range, 14 %–77 %)^37^ and nursing home residents aged ≥ 50 years in Malaysia (VE range, 55 %–76 %).^38^

In older adults, enhanced vaccine formulations have been shown to confer greater protection against influenza than their Standard-Dose (SD) counterparts. The enhanced formulations include High-Dose trivalent or quadrivalent IIVs (HD-IIV3/4), adjuvanted IIVs (aIIVs) and RIVs. A 2021 meta-analysis found that HD-IIV3 was associated with improved protection against ILI (relative VE [rVE], 15.9 %), influenza-related hospitalization (rVE, 11.7 %) and all-cause hospitalization (rVE, 8.4 %) relative to non-adjuvanted SD-IIVs.^39^ A greater reduction in mortality due to pneumonia/influenza (rVE, 39.9 %) and cardiorespiratory (rVE, 27.7 %) events was also seen with HD-IIV3.^39^ Likewise, aIIVs^40^ or RIVs^41^ also led to improved protection against influenza in older adults compared with non-adjuvanted SD-IIVs. Coleman and colleagues noted pooled rVE estimates of aIIVs against influenza-related medical encounters of 13.9 % versus SD-IIV3 and 13.7 % versus SD-IIV4. The effectiveness of allV3 in preventing influenza-related hospitalization was comparable to that of HD-IIVs.^40^ An RCT demonstrated the superiority of RIV4 to SD-IIV4 in preventing ILI among adults aged ≥ 50 years.^41^ Consequently, the Advisory Community on Immunization Practices 2023 made a preferential recommendation for HD-IIV4, aIIV4 or RIV4 over SD-IIVs for adults aged ≥ 65 years.^42^

#### Children aged 6 months to 17 years

Overall, VE estimates in children are slightly higher than those seen in adults and older adults.^28^ In particular, influenza vaccines afforded modest protection against influenza-associated hospitalization and death in children under 17.^43^^,^^44^ A 2021 meta-analysis of 37 studies demonstrated that influenza vaccination was 53.3 % effective in preventing influenza-associated hospitalization in children aged 6 months to 17 years.^43^ Interestingly, the VE estimate was higher in young children aged 6 months to 5 years than in older children aged 6–17 years (61.7 % vs. 51.7 %).^43^ Concerning different vaccine formulations, IIVs were more effective than LAIVs in preventing influenza-related hospitalization.^43^ A case-cohort analysis showed that the VE in preventing influenza-related death was 65 % among healthy children and 51 % among those with conditions that put them at high risk of influenza-related complications.^44^

Vaccinating children confers indirect protection to other individuals in the same community, school and household. A systematic review found that influenza vaccination among children aged 6 months through 17 years afforded indirect protection to members of closely connected communities against LCI, household members against LRTI or ILI, and older adults in wider communities against influenza-related mortality.^45^

#### Pregnant women and young infants

IIVs effectively reduce the risk of maternal ILI and hospitalization. A phase IV RCT of 3693 pregnant Nepali women showed that year-round vaccination with IIV3 was associated with a significant 19 % reduction in maternal febrile ILI (*p* = 0.014).^46^ A retrospective test-negative study by Thompson et al. found that vaccination during pregnancy (almost all are IIV3) was 40 % effective in reducing the risk of influenza-related hospitalization.^47^ VE estimates were similar when stratified by season timing at hospital admission and the presence of high-risk medical conditions; however, the point estimate was lower for women hospitalized in their third trimester.^47^

Influenza vaccination during pregnancy protects newborns against influenza incidence and hospitalization. The 2018 Cochrane analysis demonstrated that infants born to mothers vaccinated with IIVs were 49 % less likely to have LCI in the first 24 weeks of life,^32^ while the Nepali RCT reported a 30 % reduction in LCI incidence among infants aged 0–6 months.^46^ Another RCT revealed that infants born to IIV recipients had a 57.5 % lower risk of LRTI hospitalization in the first 3 months of life than infants born to placebo recipients.^48^ A matched case-cohort analysis found that the influenza vaccine given to pregnant women was 91.5 % effective in preventing influenza-related hospitalization among infants aged 0–6 months.^49^

#### Individuals with certain chronic medical conditions

Vaccination appears to be an important preventive tool for individuals with Chronic Obstructive Pulmonary Disease (COPD). An RCT conducted in Thailand showed that influenza vaccination was 76 % efficacious in preventing influenza-related Acute Respiratory Illness (ARI) among patients with COPD, regardless of disease severity.^50^ A recent test-negative case-control study demonstrated an average of 40 % LCI-preventive effect with current-season vaccination; VE estimates were higher for preventing outpatient cases than inpatient cases (60 % vs. 37 %).^51^ A UK-based retrospective study found that influenza vaccination during an influenza season was associated with a 41 % reduction in all-cause mortality among patients with COPD.^52^

Influenza vaccination also affords significant CV benefits to individuals with CVD. A 2021 meta-analysis of 237,058 patients with CVD showed that influenza vaccination was associated with a 25 % lower risk of all-cause mortality, 18 % lower risk of CV mortality and 13 % lower risk of Major Adverse CV Events (MACE) compared with unvaccinated controls.^53^ Importantly, IIV administration in patients with recent AMI led to a significant 28 % reduction in all-cause death, MI and stent thrombosis at 12 months (*p* = 0.040).^54^ Rates of all-cause mortality, CV mortality and MI were also lower by a respective 41 %, 41 % and 14 % among those receiving influenza vaccination.^54^ In addition, influenza vaccination is reported to have a similar range of efficacy as smoking cessation, statins or hypertensive therapy regarding the secondary prevention of acute MI.^55^

#### Vaccine safety in children aged 6 months through 17 years and adults

The different formulations of current influenza vaccines have favorable safety profiles and are generally well tolerated by most age groups. Analyses of the Vaccine Adverse Event Reporting System reports did not identify any new safety concerns following vaccination with IIVs in pediatric and adult populations, with most reported Adverse Events (AEs) being non-serious or self-limiting. Among non-death serious events, severe injection site reactions (18 %), Guillain-Barré syndrome (GBS, 13 %) and seizures (11 %) were most frequently reported following receipt of IIV4.^56^

Comparative studies found that adults aged ≥ 65 years receiving HD-IIV3 or aIIV3 experienced more local and systemic AEs than those receiving non-adjuvanted SD-IIV3.^57^^,^^58^ Among older Australian adults, reported rates of most solicited AEs were significantly higher with the receipt of HD-IIV3 than with aIIV3 or IIV4.^59^ Surprisingly, another study noted that rates of solicited local AEs (including injection-site pain and tenderness) observed within 7 days after receipt of RIV4 were significantly lower compared with IIV4.^41^

#### Vaccine safety in pregnancy

Maternal influenza vaccination did not increase the risk of adverse pregnancy and neonatal outcomes. AE incidences were similar between vaccinated and unvaccinated pregnant women in an RCT.^46^ A 2019 systematic review revealed that receipt of IIVs during pregnancy did not increase the risk of various neonatal outcomes, including small for gestational age, congenital abnormality and stillbirth.^60^ The study associated maternal influenza vaccination with reduced rates of preterm birth by 13 % (after accounting for potential confounders, such as maternal age, socioeconomic status, history of preterm birth and smoking) and low birth weight by 18 %.^60^

#### Limitations of current influenza vaccines

Influenza vaccines are most effective in seasons when the vaccine strains match the circulating strains.^30^^,^^31^ A meta-analysis of 31 RCTs with 88,468 children and adults over 26 influenza seasons revealed lower vaccine efficacy estimates during seasons with poor versus good vaccine match (59 % vs. 68 %).^30^ Similarly, an individual-participant data meta-analysis also found that influenza vaccination was more effective in preventing LCI during well-matched versus poorly matched epidemic seasons (44 % vs. 20 %) among community-dwelling older adults.^31^ As the virus constantly evolves, current influenza vaccines require reformulation each season to be effective.^4^^,^^8^

Furthermore, existing influenza vaccines only afford modest protection against influenza A and B viruses,^28^ despite a relatively high Vaccination Coverage Rate (VCR) of 51 % across all ages during the 2011–2022 season in the US.^61^ During the 2011–2022 influenza seasons, the adjusted VE of influenza vaccines in the US ranged 19 %–52 % across all age groups.^28^ Notably, influenza vaccines are less effective in older adults, with a lower adjusted VE than the general adult population (31 % vs. 36 %) during the 2011–2020 seasons.^28^

The current vaccines exhibit poor immunogenicity in individuals with compromised immunity, such as those with HIV infection. A consecutive 3-year study concluded that HIV-infected adults have impaired antibody responses to IIV3 vaccination, particularly those with low CD4+ count.^62^ Reduced immunogenicity was also observed among HIV-infected versus uninfected adults following receipt of adjuvanted vaccines.^63^ Although existing influenza vaccines generally have favorable safety profiles, a meta-analysis of 39 studies reported an association between influenza vaccination and GBS.^64^ However, several other studies did not find such an association.^65^^,^^66^

Finally, current influenza vaccines elicit antibodies mainly directed toward the highly variable globular head domain of HA glycoprotein and thus are strain specific.^8^ The use of novel approaches to induce broadly neutralizing antibodies targeting the more conserved HA receptor-binding site or the HA stem region represents the next major step towards making the universal influenza vaccine a reality.^8^^,^^67^

### WHO recommendations for influenza vaccination

Given the high mutation rate of the influenza virus, WHO continues to recommend annual influenza vaccination using the most recent vaccine formulation for high-risk individuals, including adults aged ≥ 65 years, children aged 6–59 months, pregnant women, individuals with chronic medical conditions and healthcare workers.^4^ The WHO recommends prioritizing these high-risk groups in countries considering initiating or expanding their seasonal influenza vaccination program or when vaccine supply is limited.^4^ While the WHO noted that the quadrivalent formulations are expected to provide broader protection against influenza B viruses than trivalent formulations, countries should consider whether potential health gains outweigh the costs of switching to quadrivalent vaccines.^4^

**Fig. 2:**
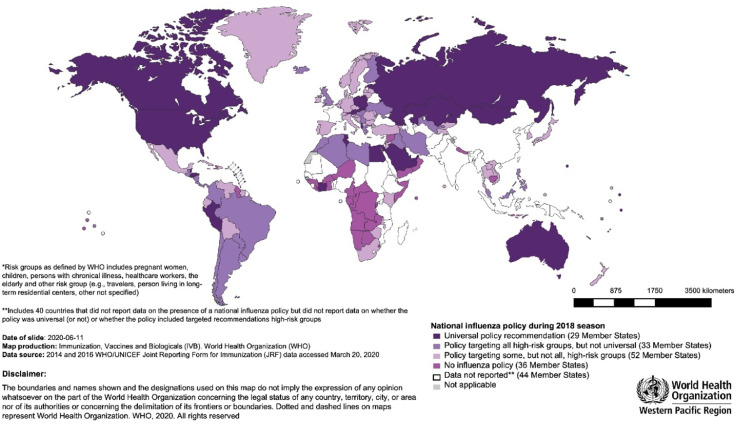
WHO member states with national influenza vaccination policies in 2018 according to risk-group prioritization. .^68^

Although the WHO recommends that all countries implement a seasonal influenza vaccination program, analysis of the WHO/UNICEF Joint Reporting Forms on Immunization in 2018 revealed a substantial variation in the execution of national influenza vaccination policies across WHO regions and World Bank income group levels.^68^ Policies were more frequently reported among HICs, while more than half of LMICs had no national policy.^68^ The Americas, Europe and Western Pacific had the highest percentages of countries with national policies, at 89 %, 89 % and 62 %, respectively. South-East Asia (27 %) and Africa (11 %) reported the lowest.^68^ Of the 56 countries that reported having a policy targeting all high-risk groups in 2018, 38 were in the Americas and Europe.^68^ HICs showed greater prioritization of all high-risk groups than LMICs (Fig. 2).^68^ Half of the countries reported using trivalent influenza vaccines in 2018, with most LMICs still preferring the trivalent formulation.^68^

Nevertheless, a recent meta-analysis estimated the pooled global VCR at only 26 % for pregnant women, 37 % for healthcare workers and 42 % for individuals with chronic diseases.^69^ The VCRs pooled from 25 countries in adults aged ≥ 60 years and children aged ≤ 14 years were 51 % and 29 %, respectively.^69^ The analysis also revealed substantial variation in VCRs among high-risk groups between countries/regions.^69^ The striking differences in VCR across WHO regions may be driven primarily by the global inequity in vaccine access.^5^

### Future of influenza vaccination

Although the past decade has seen marked improvements in the formulation of influenza vaccines, current approaches still offer moderate and variable protection and require regular reformulation.

To address the needs unmet by current vaccines, next-generation influenza vaccines should offer improved efficacy and/or effectiveness that is preferably consistent across influenza seasons, while demonstrating good safety, tolerability and acceptability profiles.^67^ Less complex manufacturing technology could help reduce the production time and also make future influenza vaccines affordable across socioeconomic strata.^26^^,^^70^

An ideal vaccine candidate should also provide long-lasting protection against the virus. Next-generation influenza vaccines should give broad protection against a wide range of influenza viruses, including group 1 or 2 influenza A, influenza B lineages or all influenza A/B viruses.^67^

#### Characteristics of an ideal influenza vaccination program

A comprehensive policy is critical to establishing a coordinated influenza vaccination program^68^ and should cover aspects such as target populations, surveillance and monitoring, education and awareness, and reimbursement. A successful vaccination program relies on adequate funding to procure sufficient vaccine doses, support trained healthcare professionals, and develop public education campaigns.^71^ Successful programs require robust healthcare infrastructure and a resilient supply chain network (from cold chain storage facilities to transportation and distribution) to ensure efficient and timely rollout.^71^

Training of healthcare workers encompasses vaccine safety and AE management, vaccine administration, vaccine handling and storage, and patient education and counselling.^72^ An effective public education campaign is crucial. Using everyday terminology in public health messaging to promote the benefits of influenza vaccination and address any safety concerns could foster vaccine confidence and reduce vaccine hesitancy.^72^^,^^73^

A multi-stakeholder partnership involving public health agencies, professional bodies and advocacy groups also plays a critical role in helping to inform vaccination policy and immunization programs and disseminate public messaging.^72^^,^^73^ Finally, the program should have robust monitoring and evaluation mechanisms to assess performance and identify areas for improvement.^72^^,^^73^

#### Learning from the success of Brazil's nationwide vaccination program

Brazil has one of the most extensive influenza vaccination programs worldwide, with VCR among at-risk groups reaching 90 % in 2019.^74^ The high VCR is driven mainly by strong government support, as influenza vaccination is fully funded under the National Immunization Program for high-risk populations.^74^ High-risk populations include adults aged ≥ 60 years, children aged 6–59 months, pregnant or postpartum women (up to 45 days), and people with chronic diseases.^74^ To raise awareness on the importance of influenza vaccination, the Brazilian Ministry of Health launches a massive public education campaign annually that typically begins in April and runs until the end of May, around the start of the influenza season.^74^

The 2019 education campaign used various online and offline media outlets to reach a broad audience.^74^ The campaign urged all high-risk citizens to receive influenza vaccination, which was provided without cost.^74^ The campaign also relied on face-to-face communication to address concerns and misperceptions about vaccination from the public.^74^ The 2019 campaign culminated in a national vaccination day, during which 41,800 mobile vaccination spots and units were set up nationwide to provide vaccination services.^74^

Good practice sharing between countries, led by regional health authorities, medical societies and community representatives, could help improve vaccination coverage and the public health impact of the program.

### Next-generation influenza vaccine technologies

New strategies to improve existing vaccine platforms have been investigated in the last few years, including nucleic acid, viral vector, and Virus-Like Particle (VLP)–based vaccines. Table 2 outlines the theoretical advantages and disadvantages of these novel technologies.^67^^,^^70^^,^^75^^,^^76^

**Table 2:** Theoretical advantages and disadvantages of the next-generation influenza vaccine platforms.^67^^,^^70^^,^^75^^,^^76^

Vaccine platform	Advantages	Disadvantages	Clinically approved examples
Viral vector vaccines	Able to mimic aspects of natural infection	Efficacy can be affected by preexisting immunity to the vector	Ebola vaccine
	Able to elicit strong immune responses without an adjuvant	Require a complex manufacturing process, affecting production time	COVID-19 vaccine
		Potential risk of genomic integration	
VLP-based vaccines	Do not require a live virus or inactivation step	Complex manufacturing process affects production time	Hepatitis B vaccine
	Non-infectious particles carrying viral antigens that can mimic live virus	Challenging to develop a novel production platform	HPV vaccine
	Able to mimic native virus conformation, with consequently improved immune responses	Stability issue during processing	
RNA vaccines	Non-infectious and non-replicating	Low stability, thus requiring low-temperature storage and transportation	COVID-19 vaccine
	Do not induce vector-specific immunity		
	Able to stimulate both innate and cellular immunity	Can elicit an interferon-mediated antiviral immune response, reducing efficacy	
	Do not interact with the host cell's DNA		
	Highly adaptable to new pathogens		
	Allows simultaneous introduction of multiple antigens		
	Ease of construction allows rapid manufacturing		
DNA vaccines	Non-infectious and non-replicating	Potential risk of genomic integration	None
	Do not induce vector-specific immunity	Poor immunogenicity in humans	
	Able to stimulate both innate and cellular immunity		
	Highly adaptable to new pathogens		
	Allows simultaneous introduction of multiple antigens		
	Stable at room temperature		
	Ease of construction allows rapid manufacturing		

COVID-19, Coronavirus Disease 2019; DNA, Deoxyribonucleic Acid; HPV, Human Papillomavirus; RNA, Ribonucleic Acid; VLP, Virus-Like Protein.

Of the novel platforms currently in clinical development (Table 3), two potential RNA vaccine candidates have recently entered phase 3 trials based on promising preliminary phase 2 results. One such trial is by Moderna, which started recruiting participants on 6 June 2022 (NCT04956575).^77^ The phase 3 trial was designed to investigate the immunogenicity and safety of the mRNA-1010 seasonal influenza vaccine in 6102 adults aged ≥ 18 years (NCT05415462).^78^ The trial was completed in September 2023.^78^ The interim analysis of the phase 2 trial showed that mRNA-1010 elicited antibody titers, consistently exceeding the 1:40 threshold level for all four strains across all vaccine doses and age groups.^77^

**Table 3:** Next-generation influenza vaccine platforms in clinical development.

	Antigenic target/MOA	Development stage	Clinical trial ID	Last update posted	Status	Trial sponsor
**RNA vaccine**						
Modified RNA	HA NAbs	Phase 3	NCT05540522; NCT05052697	27 Dec 2023; 6 Mar 2023	Active, not recruiting; completed	Pfizer
mRNA	HA NAbs	Phase 3	NCT05415462; NCT04956575	13 Sep 2023; 27 Oct 2023	Completed; completed	Moderna
		Phase 1/2	NCT05333289	7 Dec 2022	Completed	Moderna
		Phase 1/2	NCT05553301; NCT05624606	29 Sep 2023; 18 May 2023	Active, not recruiting; active, not recruiting	Sanofi
		Phase 1	NCT05446740	15 Aug 2023	Recruiting	GSK
Self-amplifying mRNA	HA NAbs	Phase 1	NCT05227001	11 Nov 2023	Completed	Pfizer
**DNA vaccine**						
Closed-circular DNA plasmid	HA NAbs	Phase 1	NCT00776711; NCT00408109; NCT00489931	2 Jul 2017	Completed	NIAID
**Viral vector vaccine**						
Alphavirus–HA	HA NAbs	Phase 1, 2	NCT00440362; NCT00706732	10 Nov 2008; 12 Jul 2012	Completed	Alphavax
Adenovirus–HA	HA NAbs	Phase 1, 2	NCT01443936	16 Dec 2019	Completed	NIAID
		Phase 1, 2	NCT01688297; NCT01335347	12 May 2017; 3 Jan 2013	Completed	VaxArt
Chimpanzee adenovirus-NP + M1	T cells	Phase 1	NCT01623518; NCT01818362	2 Dec 2014; 15 Dec 2015	Completed	University of Oxford
Modified vaccinia virus Ankara–HA	HA NAbs; T cells	Phase 1, 2b	NCT00942071	29 Nov 2012	Completed	University of Oxford
		Phase 1	NCT03277456; NCT03880474	14 Dec 2017; 26 Apr 2021	Completed; terminated	Vaccitech
**VLP-based vaccine**						
Ferritin-based, nanoparticle–HA	HA NAbs	Phase 1	NCT03186781	16 Jun 2021	Completed	NIAID
VLP–HA; NA; M1	HA NAbs; T cells	Phase 1	NCT01897701	13 Oct 2014	Completed	Novavax
Peptide–HA; NP; M1	T cells; B cells	Phase 2b (US), Phase 3 (EU)	NCT03450915; NCT02691130; NCT02293317; NCT01146119; NCT00877448	5 Oct 2021; 9 Feb 2018; 23 Feb 2016; 13 Jul 2012; 7 Mar 2023	Completed	BiondVax
Peptide–NP; M1; M2	T cells	Phase 2b	NCT02962908	16 Sep 2020	Completed	SEEK
**Recombinant protein vaccine**						
Recombinant NP	T cells; B cells	Phase 2	NCT05060887	21 Feb 2023	Completed	Osivax

DNA, Deoxyribonucleic Acid; HA, Hemagglutinin; M1, Matrix 1 protein; M2, Matrix 2 protein; mRNA, Messenger RNA; MOA, Mode Of Action; NA, Neuraminidase; NAb, Neutralizing Antibody; NIAID, National Institute of Allergy and Infectious Diseases; NP, Nucleoprotein; RNA, Ribonucleic Acid; VLP, Virus-Like Protein.

Pfizer/BioNTech also commenced their phase 3 trial in 2022, following the readout of the interim phase 2 trial results. The phase 2 trial showed that, in healthy adults aged ≥ 65 years, quadrivalent modified mRNA (modRNA) induced substantially greater CD4+ and CD8+ responses to all four influenza strains than the licensed SD-IIV4 (NCT05052697).^79^ The phase 3 study was designed to evaluate the efficacy, safety and tolerability of modRNA in 46,180 healthy adults (NCT05540522).^80^ At the time of this writing, the phase 3 trial is still active but no longer recruiting; it is expected to be completed by March 2024.^80^

## Summary

While indispensable in the fight against influenza, existing vaccines have several limitations. New vaccine technologies are urgently needed to overcome shortcomings of the currently available influenza vaccine. In particular, RNA vaccine technology offers several advantages over other approaches, such as a favorable safety profile. RNA is a non-infectious, non-replicating and non-integrating molecule. It also does not induce vector-specific immunity. Importantly, the ease of production allows rapid and scalable production, potentially making it cost-effective. Notably, the mRNA vaccine platform can be designed to target multiple antigens, allowing for the development of a universal influenza vaccine candidate or a combination vaccine candidate against several respiratory viruses. Recently, two influenza mRNA vaccine candidates have entered phase 3 clinical trials, emerging as promising alternatives to conventional technologies.

## Conflicts of interest

The authors declare no conflicts of interest.
